# YM155 potently kills acute lymphoblastic leukemia cells through activation of the DNA damage pathway

**DOI:** 10.1186/s13045-015-0132-6

**Published:** 2015-04-22

**Authors:** Bill H Chang, Kara Johnson, Dorian LaTocha, Joelle S J Rowley, Jade Bryant, Russell Burke, Rebecca L Smith, Marc Loriaux, Markus Müschen, Charles Mullighan, Brian J Druker, Jeffrey W Tyner

**Affiliations:** Division of Pediatric Hematology and Oncology, Department of Pediatrics, Oregon Health & Science University, Portland, OR 97239 USA; OHSU Knight Cancer Institute, Portland, OR 97239 USA; Department of Pathology, Oregon Health & Science University, Portland, OR 97239 USA; Department of Laboratory Medicine, University of California San Francisco, San Francisco, CA USA; Department of Oncology, St Jude Children’s Research Hospital and University of Tennessee Health Science Center, Memphis, TN 38105 USA; Howard Hughes Medical Institute, Portland, OR 97239 USA; Department Cell & Developmental Biology, Oregon Health & Science University, Portland, OR 97239 USA

**Keywords:** Acute lymphoblastic leukemia, YM155, DNA damage

## Abstract

**Background:**

Novel-targeted therapies are in rapid development for the treatment of acute lymphoblastic leukemia (ALL) to overcome resistance and decrease toxicity. Survivin, a member of the inhibitor of apoptosis gene family and chromosome passenger complex, is critical in a variety of human cancers, including ALL. A well-established suppressor of survivin has been the small molecule, YM155. Reports are identifying other mechanisms of action for YM155. Therefore, we sought to investigate the mode of action and role of YM155 for therapeutic use in the context of ALL.

**Methods:**

Primary ALL samples and ALL cell lines were interrogated with YM155 to identify drug sensitivity. Ph^+^ALL harboring the *BCR-ABL1* oncogene were tested for any interaction with YM155 and the multi-kinase inhibitor dasatinib. Representative ALL cell lines were tested to identify the response to YM155 using standard biochemical assays as well as RNA expression and phosphorylation arrays.

**Results:**

ALL samples exhibited significant sensitivity to YM155, and an additive response was observed with dasatinib in the setting of Ph^+^ALL. ALL cells were more sensitive to YM155 during S phase during DNA replication. YM155 activates the DNA damage pathway leading to phosphorylation of Chk2 and H2AX. Interestingly, screening of primary patient samples identified unique and exquisite YM155 sensitivity in some but not all ALL specimens.

**Conclusion:**

These results are the first to have screened a large number of primary patient leukemic samples to identify individual variations of response to YM155. Our studies further support that YM155 in ALL induces DNA damage leading to S phase arrest. Finally, only subsets of ALL have exquisite sensitivity to YM155 presumably through both suppression of survivin expression and activation of the DNA damage pathway underscoring its potential for therapeutic development.

**Electronic supplementary material:**

The online version of this article (doi:10.1186/s13045-015-0132-6) contains supplementary material, which is available to authorized users.

## Background

B-cell precursor acute lymphoblastic leukemia (ALL), the most common pediatric malignancy, affects all age groups and carries a high treatment burden. Tremendous strides have been successful in improving the treatment and cure of this disease (review [1]). Unfortunately, standard cytotoxic treatment continues to expose the patient to long-term morbidity and mortality. Therefore, novel-targeted therapies will be essential to decrease therapeutic burden of ALL. To that end, multiple targets have recently been identified and novel therapies are now being tested both in preclinical and clinical studies [2].

Survivin (*BIRC5*) is a small protein that belongs to the inhibitor of apoptosis (IAP) family and chromosome passenger complex (review [3,4]). Survivin is an attractive target for therapy because it is expressed predominantly during development and in the setting of malignancies with little to no expression in terminally differentiated tissue [5-7]. Further, survivin overexpression has correlated with resistant and refractory disease in pediatric ALL [8] while suppressing expression in ALL decreases chemoresistance [9,10]. Although several mechanisms of survivin inhibition have been studied, early trials have shown minimal success in targeting this protein for clinical benefit [11].

YM155 (sepantronium bromide) is a small molecule originally discovered as a suppressant of survivin expression [12]. YM155 has shown potent antiproliferative effects on a variety of human cancer cell lines [13]. Although no human trials have been designed to test this drug in ALL, it has theoretical potential for clinical benefit. We recently verified the utility of YM155 in decreasing survivin expression in pediatric ALL [14]. These studies also found that survivin suppression by YM155 was not the sole effect that caused cell death. Specifically, in primary ALL patient samples, sensitivity did not correlate with survivin expression. In fact, other investigators have shown that YM155 has the potential to cause cell death by mechanisms other than survivin repression in different disease models [15-18]. One intriguing concept is that YM155 has the potential to cause DNA damage thereby increasing cell death [15,18]. In the following studies, we used a rapid functional assay on fresh primary leukemic samples to identify sensitivity to YM155 and further validate the mechanism of action of YM155 in the setting of ALL.

## Results

### Primary patient ALL samples are significantly more sensitive to YM155 as compared to primary patient AML samples

**Figure 1:**
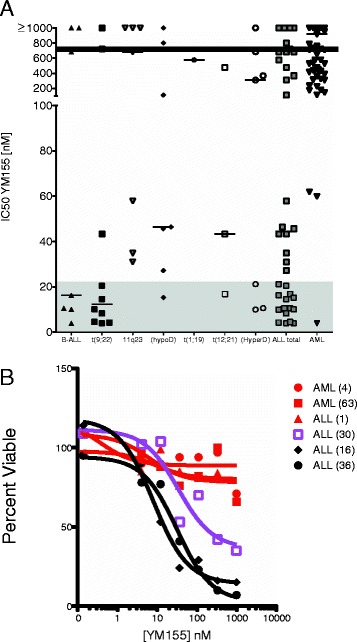
Response to YM155 of primary ALL and AML patient samples. Primary patient and xenografted samples were collected as previously described [14]. **(A)** Samples were then incubated with increasing concentrations of YM155 (0 nM to 1 μM) and IC_50_ were calculated using a second-order polynomial. (Filled triangle) ALL samples without a recurring cytogenetic abnormality; (filled diamond) ALL with t(9;22); (filled circle) ALL with 11q23 rearrangement or MLL rearrangement; (filled square) ALL with <44 chromosomes or hypodiploid; (open triangle) ALL with t(1;19); (open square) ALL with t(12;21); (open circle) ALL with hyperdiploid; (grey square) total ALL; (grey triangle) total AML samples. Statistical significance of *p* < 0.05 by Student’s *t*-test between total ALL and AML samples. Samples with IC_50_s within the shaded area are within levels achievable in phase 1 pharmacokinetics [30]. Horizontal line denotes median IC_50_ of all tested samples. **(B)** Dose–response curves of two AML-resistant samples (red filled circle, red filled square), one ALL resistant sample (red filled triangle), one ALL intermediate sample (purple open square), and two sensitive ALL samples (black filled diamond, black filled circle). Numbers in parentheses denote patient number (Additional file 2: Table S1 and S2).

We have recently developed a technique to assess sensitivity to small molecule inhibitors from primary patient leukemic samples obtained at diagnosis [19]. Using this platform, we interrogated over 40 ALL and 65 AML samples for sensitivity to YM155 (Figure 1A). These primary patient samples exhibited a diverse range of sensitivity to the drug. For each subgroup, the number of samples within the subgroup, the median IC_50_, mean IC_50_, standard deviation, and standard error are shown in Table 1.The ALL samples appeared to have a bimodal distribution of IC_50_s. One group showed significant sensitivity while another group showed a relative resistance to the drug. Due to this distribution, the median IC_50_ for the ALL samples was calculated to 45 nM (Table 1). In contrast, the majority of AML samples showed minimal sensitivity to YM55 with a calculated median IC_50_ of 900 nM. Sensitive samples showed robust inhibition of viability whereas resistant samples showed no toxicity from YM155 up to 1 μM (Figure 1B). One ALL subgroup, samples with t(9;22)/*BCR-ABL1* (Ph^+^ALL), appeared quite sensitive to YM155, though the sample size of each genetic subgroup was too small to achieve statistical significance (Figure 1A).

**Table 1:** **Number of samples within each subgroup, the median IC**_**50**_ **, mean IC**_**50**_ **, standard deviation, and standard error**

**Disease**	**B-ALL**	**t(9;22)**	**11q23**	**hypoD**	**t(1;19)**	**t(12;21)**	**hyperD**	**ALL total**	**AML**
Number	7	10	7	7	1	3	7	42	69
Median	16.31	12.34	684.9	46.45	576.4	43.41	310	44.54	918.3
Mean	389.4	183.1	544.1	292.2	576.4	179	343.6	331.6	712.4
Std. deviation	484.4	363.3	483.4	420.3	0	258.3	381.9	406.3	327.9
Std. error	183.1	114.9	182.7	158.9	0	149.1	144.4	62.69	39.48

### YM155 shows synergy with dasatinib in Ph^+^ALL

**Figure 2:**
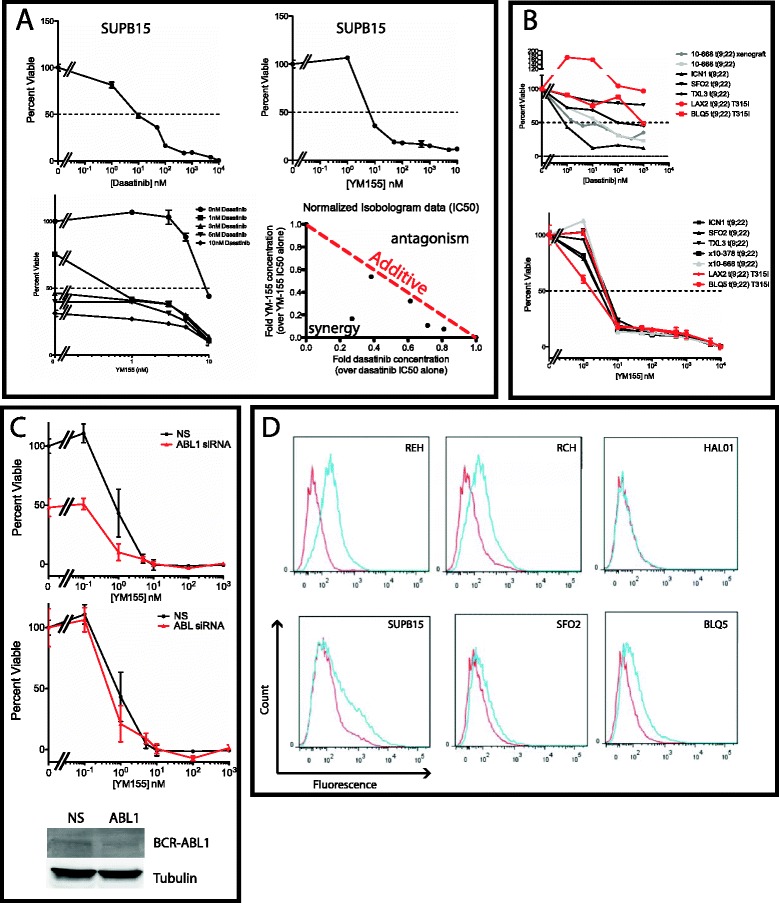
Ph^+^ALL samples are sensitive to YM155. **(A)** The Ph^+^ALL cell line SUPB15 was tested for sensitivity to dasatinib (top left) and YM155 (Top right) with increasing concentrations of drug (0 nM to 10 μM). The IC_50_ for each drug was approximately 10 nM. Combination of YM155 and dasatinib on SUPB5 cells showed a decrease in IC_50_ suggestive of synergy/additive by isobologram analysis [34]. Dose–response of SUPB15 cells with YM155 was carried out with increasing concentrations of dasatinib (bottom left). IC_50_ of individual drug alone was used as the reference value of 1 and subsequent IC_50_ of combination of drugs were compared (bottom right). Points on the red dotted line would be additive, while points left of the line would be synergistic and points on the right would be antagonistic. **(B)** Sensitivity to YM155 and dasatinib was further tested on primary Ph^+^ALL patient samples (10–668) and xenograft samples (10–668 xenograft, ICN1, SFO2, TXL3, LAX2, BLQ5, x10-378). Red samples signify dasatinib-resistant T315I mutants. **(C)** Knockdown of BCR-ABL1 expression. SUPB15 were treated with either non-specific (NS) or ABL1 siRNA. The cells were then incubated in increasing concentrations of YM155 (0 to 1 μM) for 4 days and then assayed for viability with MTS. Top panel describes the viability of the cells normalized to NS without YM155 exposure. Bottom panel describes the viability normalized to with NS or ABL1 without YM155 exposure. An aliquot of electroporated cells were used for immunoblot analysis of BCR-ABL1 knockdown 3 days after electroporation. **(D)** P53 Ser-15 phospho-flow after treatment with YM155. Each cell line (REH, RCH, HAL01, and SUPB15) and xenograft samples (SFO2, BLQ5) were treated with 100 nM YM155 for 24 h. (Red peak) Control signal for ser15-phospho-p53. (Blue peak) YM155 treatment. REH and RCH cells showed a distinctive increase in phosphorylation of p53. In contrast, the Ph^+^ALL cells showed minimal increase in p53 phosphorylation.

Our previous report supported the concept of inhibiting survivin expression in Ph^+^ALL as a therapeutic option [14]. Since these specimens harbor a known oncogene (e.g., *BCR-ABL*) for which targeted agents are available (e.g., imatinib, dasatinib), we next wanted to test whether YM155 would have benefit in combination with dasatinib. Indeed, there appeared to be additive/synergy with this combination (Figure 2A). Further, YM155 sensitivity appeared to be maintained in primary Ph^+^ALL samples, xenografted samples, and in samples with dasatinib-resistant mutations (Figure 2B). To further address the combination of dasatinib and YM155, we performed knockdown experiments using siRNA to ABL1. Reduced expression of BCR-ABL1 did not enhance or reduce sensitivity to YM155 suggesting an additive effect of YM155 and dasatinib (Figure 2C).

We next wanted to validate our previous data that showed that inhibition of survivin expression would lead to phosphorylation of p53 [14]. To determine whether sensitivity to YM155 is universally related to p53 activation, we developed a phosphoflow assay using ser15 phospho-p53 (Figure 2D). Both REH and RCH cell lines showed robust enhancement of phospho-p53 signal. In contrast, Ph^+^ALL cell lines and xenograft samples SFO2 and BLQ5 showed minimal increase of p53 phosphorylation using a dose closer to an IC_90_ of 100nM, despite both lines showing sensitivity to YM155. These results suggest alternative mechanisms for YM155 sensitivity beyond p53 activation.

### YM155 causes an S phase arrest in ALL cells

**Figure 3:**
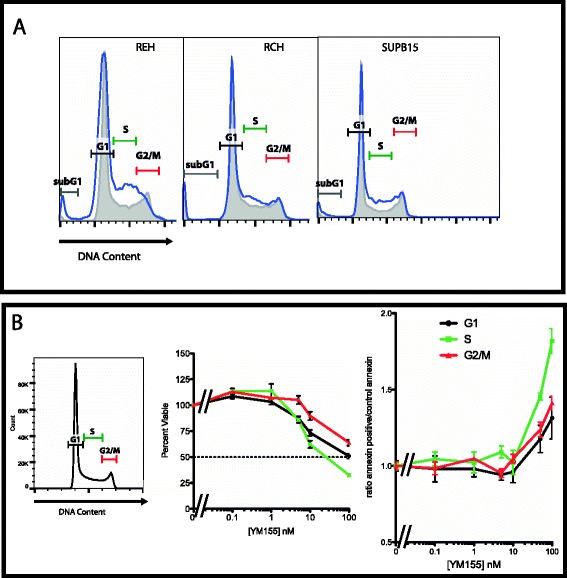
YM155 causes an S phase arrest. **(A)** Cell cycle arrest with YM155 treatment. An asynchronous population of cells (REH, RCH, and SUPB15) were treated with 100 nM YM155 for 24 h and assayed for DNA content by propidium iodide. (Grey line filled) Control asynchronous population. (Blue line) YM155 treatment. Bars indicate the cell cycle (subG1, G1, S, G2/M). All three cell lines show an increase in S phase and a slight increase in the sub G1 phase within 24 h of treatment. **(B)** YM155 treatment has a greater effect when exposed to cells that are in S phase of the cell cycle. An asynchronous REH population was sorted by DNA content for G1, S, and G2/M and treated with increasing concentration of YM155 (0 to 100 nM) for 24 h, then assessed for viability using an MTS colorimetric assay (middle panel) or for Annexin V staining (right panel).

The unexpected results of our p53 phospho-flow assay led to the possibility that YM155 may not be activating p53 through survivin suppression alone. To begin to test this hypothesis, we chose to interrogate what effects YM155 had on the cell cycle. Survivin expression has been closely linked with the cell cycle with minimal expression until G2/M [20], suggesting that a reduction in survivin levels would result in a G2/M arrest. Yet, previous studies suggest that YM155 has effects on survivin expression independent of the cell cycle [21]. In contrast to prior published results, we did find a subtle increase in the number of cells in S phase when treated with 100 nM YM155 for 24 h in REH, RCH, and SUPB15 cells (Figure 3A). Further, we see a slight increase in the subG1 population consistent with an increase in apoptotic cells (Table 2).

**Table 2:** **Percent of subpopulations within the cell cycle with YM155 treatment**

	**subG1 (%)**	**G1 (%)**	**S (%)**	**G2/M (%)**
REH				
Control	1	61	16	21
YM155	3	61	20	13
RCH				
Control	4	62	19	15
YM155	6	55	24	15
SUPB15				
Control	2	63	21	13
YM155	5	51	31	10

These findings would suggest that YM155 can cause an S phase arrest supporting effects during DNA replication. To determine the stage of the cell cycle where YM155 has the most significant effects, we stained a live asynchronous population of REH cells with either Hoescht or Vibrant DyeCycle Violet and sorted for cells in G1, S, or G2/M phase. The sorted cells were plated in increasing concentrations of YM155 (0 to 100 nM) for 24 h and tested for viability with MTS and apoptosis with annexin V staining. The cells in S phase appeared to be significantly more sensitive to YM155 at these doses by both decrease in cell viability by MTS and increase in early apoptosis by annexin V staining (Figure 3B). Taken together, these data suggest that YM155 has significant effects on the cells in S phase, a time of minimal expression of survivin [14]. Therefore, YM155 may have other effects independent of its ability to suppress expression of survivin in ALL.

### YM155 has effects on gene expression and phosphorylation

**Figure 4:**
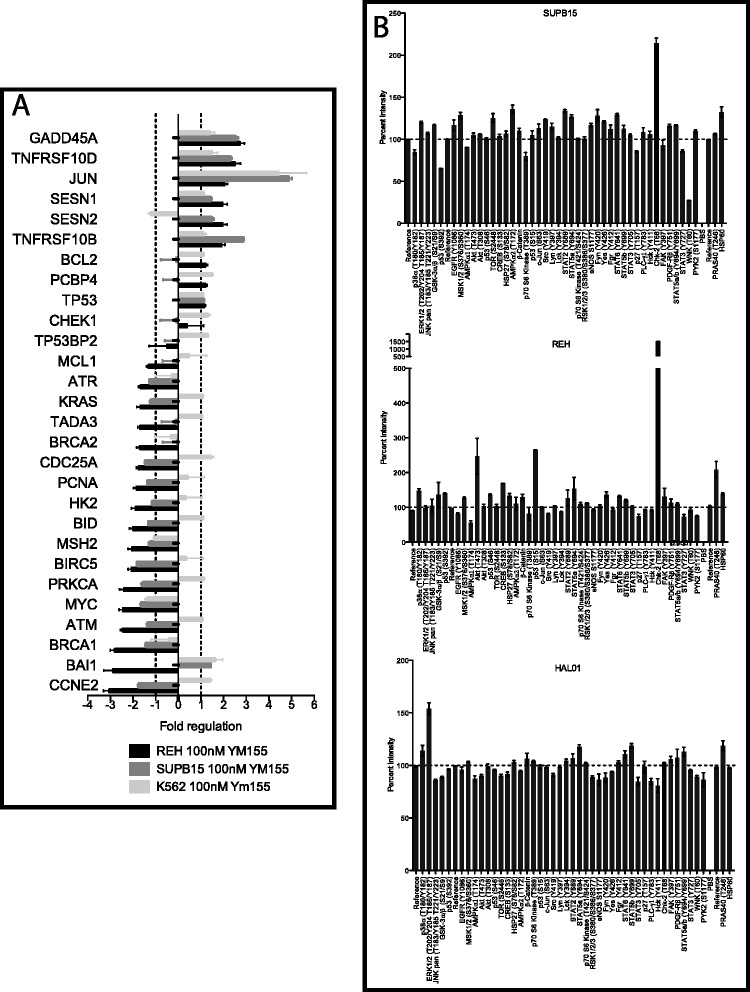
YM155 activates DNA damage response. **(A)** YM155 has multiple effects on RNA expression. REH (wild-type p53), SUPB15 (wild-type p53), and K562 (mutant p53) cells were treated with 100 nM YM155 or vehicle for 24 h and mRNA expression levels of 84 genes were evaluated using the P53 RT^2^ Array. Treatment with YM155 caused about a twofold decrease in survivin mRNA (*BIRC5*) in the p53 intact cells, but had minimal impact on survivin expression in K562 cells despite potent effects of YM155 on K562 cell growth/viability (IC_50_ of <10nM [13]). Meanwhile, other genes such as *JUN* and *GADD45A* involved in DNA damage response exhibit increased expression after YM155 treatment in all three cell lines. **(B)** YM155 treatment greatly enhances phosphorylation of Chk2. REH, SUPB15, and HAL01 cells were treated with either vehicle or 100 nM YM155 for 24 h, and protein phosphorylation patterns were assessed using Proteome Profiler Arrays. Values were quantified and normalized to untreated control for each site. REH cells show p53 and Chk2 with the largest change in phosphorylation. SUPB15 shows only Chk2 with the largest change in phosphorylation. HAL01 cells, known to be resistant to YM155 showed minimal change in phosphorylation.

Studies have argued that other genes such as *MCL1* can be downregulated by YM155 (Additional file 1: Figure S1 and [22]). In order to determine what other genes may play a role in YM155 sensitivity, we used the p53 RT^2^ Array (84 genes). This assay allowed us to evaluate gene expression changes of 84 genes after a 24-h treatment of asynchronous cells with 100 nM YM155, including survivin and Mcl1. We identified a variety of genes that exhibited at least a twofold change in mRNA expression level after exposure to YM155 (Figure 4A). Two p53 wild-type cell lines REH and SUPB15 showed a twofold decrease in survivin (*BIRC5*) expression. Both cell lines show similar decreases in other genes such as *BRCA1* and *CCNE2.* The p53 mutant cell line K562, which is quite sensitive to YM155 [13], showed virtually no change in survivin expression. In all three cell lines, genes known to be involved in DNA damage response, such as *GADD45A* and *JUN* [23], were upregulated suggesting that YM155 may induce more global effects on the cells through DNA damage.

Since our previous studies showed that p53 phosphorylation increases with YM155 treatment [14], yet p53 mutant cells are still sensitive to YM155, we chose to identify other signaling pathways that are affected by YM155 treatment. ALL cell lines were treated with 100 nM YM155 for 24 h, then harvested and assessed for changes in phosphorylation using a phospho-proteome array (Figure 4B). As seen in our phospho-flow assay, REH cell showed a significant impact of YM155 on p53 phosphorylation while SUPB15 cells showed minimal increase in p53. Instead, both cell lines showed a dramatic increase in Chk2 at (Thr68). HAL01 cells, known to be resistant to YM155, showed minimal change in phosphorylation. These results would identify Chk2 phosphorylation as a downstream effect of YM155 treatment.

### YM155 increases phospho-Chk2 and direct DNA damage

**Figure 5:**
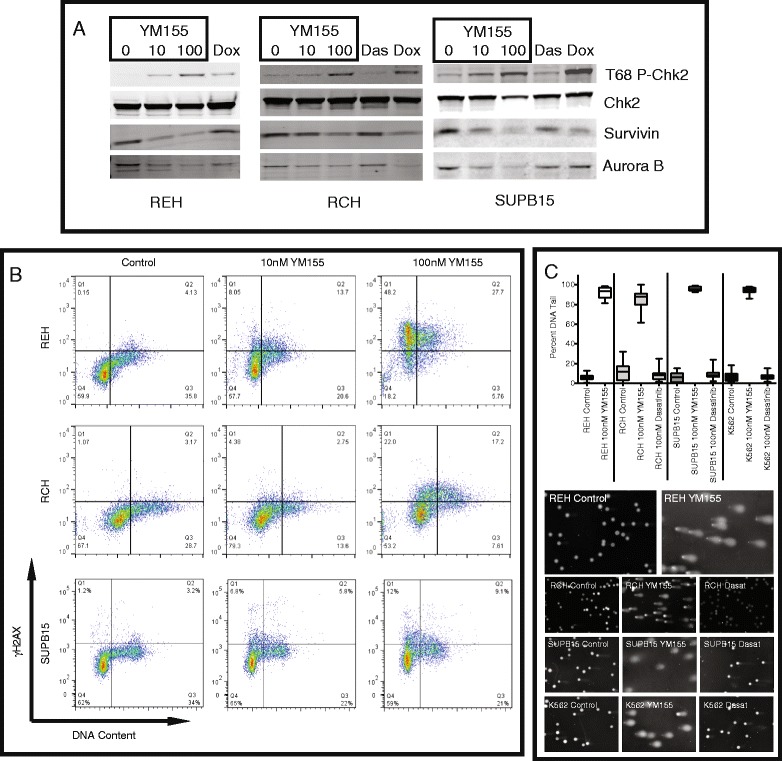
YM155 activates DNA damage response with S phase arrest. **(A)** Immunoblot verifies the increase in threonine 68 phosphorylation of Chk2. REH, RCH, and SUPB15 cells were treated with increasing concentrations of YM155 (0, 10, 100 nM), doxorubicin (0.1 μg/ml) or dasatinib (100 nM) for 24 h. Whole cell lysates were subjected to immunoblot using antibodies specific for phospho-T68 Chk2, total Chk2, survivin, and Aurora B kinase. **(B)** Treatment with YM155 causes an increase in γH2AX. REH, RCH, and SUPB15 cells were treated with vehicle or YM155 (10 and 100nM) for 24 h and then stained with γH2AX-FITC and Vibrant DyeCycle Violet Stain. The cells were quantified using FACS/AriaIII for DNA content and FITC. Treatment with YM155 in each cell line showed a dose-dependent increase in γH2AX-FITC, mostly in G1 and S. **(C)** Comet assays of leukemia cell lines identify DNA damage. REH, RCH, SUPB15, and K562 cells were treated with 100 nM YM155 and 100 nM Dasatinib (excluding REH) for 24 h. Top panel is the box-whisker plot of the percent of DNA in the comet tail after treatment. Bottom panels are photographic representations of the comet assays for each cell line.

These studies point to the possibility that YM155 induces a DNA damage response during S phase. Previous reports have also implied that the structure of YM155 has the potential to cause DNA damage similar to chromomycin A3, bisantrene HCl, and actinomycin D [15,18]. To validate our results from the phospho-proteome array, ALL cells were treated with YM155 with 10 and 100 nM for 24 h and immunoblotted for phospho-T68 Chk2, total Chk2, and survivin (Figure 5A). As another marker for the cells in G2/M, the extracts were immunoblotted for Aurora B kinase. In addition to YM155, the cells were also treated with a known DNA damaging agent doxorubicin and with dasatinib. REH, RCH, and SUPB15 cells all show a dose-dependent increase in phospho-T68 Chk2 in support of the proteome results. Doxorubicin treatment shows a similar increase in Chk2 phosphorylation. Although there is a dose-dependent decrease in survivin, there is also a decrease in Aurora B kinase expression suggesting the decrease in survivin may be due in part to an S phase arrest. In contrast, dasatinib does not significantly impact either phosphorylation or expression.

Upon sensing DNA damage, the phosphatidylinositol 3′-*kinase*-related *kinases* (PIKKs) become activated and phosphorylate three important substrates, p53 (at serine 15), Chk2 (at threonine 68), and histone H2AX (at serine 139) [24]. We have already identified that p53 and Chk2 are phosphorylated when exposed to YM155. To test whether H2AX is phosphorylated at serine 139 (γH2AX), we treated YM155-sensitive cell lines with 10 and 100 nM YM155 for 24 h, partially fixed and assayed for DNA content and γH2AX (Figure 5B as described in [25]). Each cell line showed a dose-dependent increase in γH2AX staining in G1 and S phase. To further test for DNA damage, we performed comet assays after treatment with 100 nM YM155 for 24 h (Figure 5C). REH cells showed significant damage after treatment. Further, RCH, SUPB15 cells, and the p53 mutant K562 cells also showed significant DNA damage from YM155 with minimal change after 24-h treatment with 100 nM dasatinib. These results confirm that YM155 activates the DNA damage pathway independent of p53 activity.

## Discussion

Molecular targeting of survivin has been an intriguing concept for therapy. Unfortunately, it has been a difficult protein to target as this protein has no known catalytic activity (review [3]). One of the compounds with the most preclinical and clinical data is YM155. YM155 was originally identified as a specific inhibitor of survivin expression from a high throughput screen using a survivin promoter luciferase assay [12]. Since its first description, there have been multiple studies that have validated this compound as being selective for survivin expression [13,21,26]. There is now emerging data that YM155 may have more off-target effects causing cell death, including inhibition of Mcl1 expression and direct DNA damage [15,17,22]. Our studies further validate that YM155 has the potential to activate the DNA damage response through an S phase arrest that can increase p53, Chk2, and H2AX phosphorylation eventually leading to apoptosis. These results, consistent with prior studies showing direct DNA damage by intercalation [18], are the first to be described in acute lymphoblastic leukemia cells. Further, our results are the first to have screened a variety of primary hematologic malignancies showing a significant amount of heterogeneity. As DNA damaging agents are a mainstay of therapy for ALL, it is critical that these studies highlight the exquisite sensitivity to YM155 and the mechanisms of action in ALL.

Prior studies have suggested other DNA damaging agents such as doxorubicin increase survivin expression in p53 intact leukemic cells [27]. In those studies, an intriguing concept of combination therapy with doxorubicin and survivin suppression was proposed. Further studies identified that YM155 can further inhibit radiation-induced DNA repair [28]. Although the authors ascribed this phenomenon to inhibition of survivin expression, another possibility would be that YM155 was further inducing DNA damage.

DNA damaging agents such as doxorubicin is a mainstay in the treatment of leukemia. It is a highly effective drug but carries with it both significant short-term and long-term side effects including myelosuppression and cardiac toxicity. Our studies would suggest at least two possibilities. First, in future clinical trials testing YM155, concurrent use of DNA damaging agents such as doxorubicin may be discouraged for possible compounding of toxicity. Second, if YM155 is shown to have some efficacy in pre-selected patients, future studies could be designed to substitute YM155 for anthracyclines which may decrease long-term side effects.

One of the most intriguing results of these studies is the heterogeneity of sensitivity of primary patient samples. Our study is the first to have screened a multitude of primary patient samples. Overall, a subset of ALL samples appears to be quite sensitive compared to AML samples. Further, all tested subtypes such as hypodiploid, MLL-rearranged, and Ph^+^ALL have samples that are quite sensitive while others remained resistant. Unfortunately, our sample size was not powered to find a correlation between sensitivity and current prognostic features such as cytogenetic subtypes. Instead, our studies highlight the power of a functional assay to predict individual patients that could respond to drug. This heterogeneity of response may explain in part why current clinical trials testing YM155 have shown minimal success. Our functional results predict that individuals need to be preselected for YM155 sensitivity. Only then could a trial show benefit from testing this drug.

Our previous results from primary patient samples did not find a correlation with survivin expression and YM155 sensitivity [14]. One intriguing possibility for this heterogeneity lies in the expression of the solute carrier SLC35F2 [18]. Their studies suggest that the DNA damage sensitivity by YM155 correlates with expression of SLC35F2. Although these studies did not interrogate leukemic samples, their findings may address in part the heterogeneity seen in leukemia. For example, ours studies showed that the HAL01 cell line appeared the least sensitive to YM155. This cell line and primary ALL with the t(17;19) have previously been shown to have higher expression of the efflux protein ABCB1 [29], suggesting drug efflux as an important mechanism of resistance. Therefore, future studies will be needed to identify a specific marker to predict *in vitro* response to YM155 in ALL samples.

In the original article by Nakahara et al., they described a broad distribution of sensitivities to YM155 in malignant cell lines tested including many solid tumors [13]. In their orthotopic animal studies, YM155 treatment suppressed tumor growth on samples with GI_50_ of 5–20 nM. In these animal studies, YM155 was well tolerated with minimal side effects at therapeutic doses. Further, in previous phase 1 clinical trials with YM155, plasma concentrations of approximately 25 nM were achieved that were also well tolerated with minimal side effects [30]. These inhibitory concentrations are well within the IC_50_s of our sensitive primary patient samples predicting the possibility of *in vivo* response to YM155.

Current clinical trials testing the efficacy of YM155 as a single agent and in combination with either immunotherapy or cytotoxic chemotherapy have verified that the drug is quite tolerable in these scenarios (review [31]). Unfortunately, there have been very minimal responses. Our data supports the concept of YM155 as an excellent candidate drug to add to therapeutic regimens only in a subgroup of patients that have a pre-selected possibility of responding. This includes patients with Ph^+^ALL who may benefit from combination therapy. Our concept would be as a combination for patients with recurrent ALL that have been shown to be sensitive *in vitro* as selection to enroll on the therapeutic trial. The caution is that because of the heterogeneity of the drug response, future trials testing YM155 in patients need to pre-select the sensitive population to validate its efficacy.

## Conclusions

Our studies are the first to interrogate a significant number of primary leukemic samples for functional sensitivity to YM155. These results show the potential benefit of rapid functional preselection of patients that could potentially respond to drug. Further, we identify that ALL has a unique subset that is exquisitely sensitive to this drug. The ALL cell lines further identify that YM155 not only reduces survivin expression but also activates DNA damage pathways consistent with other DNA damaging agents. Future studies will be needed to identify the mechanism of selective sensitivity of YM155 in ALL to select patients that would have the capacity of responding to this drug for therapeutic effect.

## Methods

### Chemicals and reagents

Fetal bovine serum was obtained from Hyclone (Thermo Scientific, Rockford, IL). All other tissue culture reagents were obtained from Invitrogen (Grand Island, NY). Viability assays were performed with CellTiter 96 AQueous One solution cell proliferation assay (MTS) from Promega (Madison, WI). YM155 was purchased from Selleck (Houston, TX) and solubilized in dimethyl sulfoxide at 10 mM stock. Dasatinib was purchased from LC labs (Woburn, MA) and also solubilized in dimethyl sulfoxide at 10 mM stock. Graphical and statistical data were generated using either Microsoft Excel or GraphPad Prism.

### Cell lines and tissue culture

RCH-ACV (RCH) (DSMZ, Braunschweig, Germany) is a pediatric BCP-ALL cell line from a patient with recurrent disease carrying the *E2A-PBX1* t(1;19) chimeric protein. REH (ATCC, Manassas, VI) is a pediatric ALL cell line from a patient with recurrent disease carrying the *ETV6-RUNX1* t(12;21) chimeric protein. SUPB15 (ATCC) is a pediatric ALL cell line also from a patient with recurrent disease carrying the *BCR-ABL* t(9;22) translocation. HAL01 cells (DSMZ) are from a pediatric patient with *de novo* ALL with the *E2A-HLF* t(17;19)*.* K562 (ATCC) is a chronic myeloid leukemia cell line that also carries the *BCR-ABL* t(9;22) translocation. RCH, REH, HAL01, and K562 cells were maintained in RPMI with 10% FBS, 4 mM glutamine, and 1% penicillin and streptomycin. SUPB15 cells were maintained in RPMI with 20% FBS, 4 mM glutamine, 50nM 2-mercaptoethanol, and 1% penicillin and streptomycin. All cell lines were originally obtained from their respective cell banks (DSMZ or ATCC) and were authenticated by standard RT-PCR for their respective translocations.

No human subjects were directly involved in the research. Biological samples were obtained with written informed consent. Procurement of biological samples was approved by the Institutional Review Board of Oregon Health and Science University (IRB #4422). Patient sample characteristics are defined as in Additional file 2: Tables S1 and S2.

Primary ALL xenograft samples were obtained from the lab of Markus Müschen. Briefly, primary patient samples were injected into immunodeficient NOD/SCID mice and expanded. Leukemic cells were then harvested from the spleen, and mononuclear cells were isolated by ficoll gradient and used for subsequent drug testing.

### Drug treatment and viability assay

ALL cell lines (5,000 cells per well) and primary patient samples (50,000 cells per well) were incubated with increasing concentrations of YM155 (0 to 10 μM) or dasatinib (0 to 10 μM) in RPMI with 10% FBS. After 3 days, cells were subjected to MTS for assessment of cell viability. All values were normalized to the no drug control from each respective cell line.

### siRNA treatment

SUPB15 cells (800,000 cells per treatment) were incubated with 40 μM siRNA (Dharmacon) of either non-specific (NS) or ABL1 in siPORT^TM^ siRNA electroporation buffer (Life Technologies), then electroporated using GenePulser Xcell (BioRad).

### P53 phosphoflow

ALL cells were treated for 24 h with 100 nM YM155 and fixed with 1% formaldehyde. The cells were then stained with (Ser 15) phospho-p53 Alexa 488 (Cell Signaling, Beverly, MA) in PBS/1% BSA. The cells were then subjected to flow cytometry with BD FACS/Aria and interpreted by FlowJo (TreeStar, Ashland, OR).

### DNA content flow sort

ALL cell lines were grown in RPMI/10% FBS to a concentration of 5–10 × 10^5^ cells/ml. The cells were then washed with PBS/1%FBS and stained with Hoescht 33342 (Life Technologies) at 5 μg/ml or Vibrant DyeCycle Violet at 5 μM (Molecular Probes) for 30 min at 37°C. The cells were then sorted by DNA content and then treated with graduating concentrations of YM155 (0–100 nM YM155) for 24 h. After treatment, the cells were subjected to MTS for assessment of cell viability and Annexin V staining (Guava Nexin) for activation of apoptosis. ALL cell lines were also treated with 100 nM YM155 for 24 h, then washed with PBS and stained with buffer containing 3 mM EDTA, pH 8.0, 0.05% NP-40, 50 μg/ml propidium iodide, and 1 mg/ml RNAse A in PBS [32].

### Expression arrays

P53 RT^2^ Array (QIAGEN, Germantown, MD) were purchased and used according to the manufacturer’s guidelines. ALL cell lines were subjected to 100 nM YM155 for 24 h, and RNA was extracted from the cell line using RNAeasy (QIAGEN). One microgram of RNA was used to make first strand cDNA using RT^2^ First Strand kit. Quantitative PCR was performed on Opticon 2 (MJ Research). Generated data was then uploaded to the RT^2^ Array website.

### Phospho proteome assay

Proteome Profiler Arrays (R&D systems, Minneapolis, MN) were purchased and used according to the manufacturer’s guidelines. ALL cell lines were subjected to 100 nM YM155 for 24 h, and 1.2 μg of total protein was incubated per assay. Results were visualized by chemiluminescence using a Lumi-Imager (Boerhinger Mannheim) with densitometry performed with ImageJ 1.64.

### Immunoblot analysis

The cells were washed with phosphate buffered saline (PBS) and lysed with 1× sodium dodecyl sulfate (SDS) loading buffer (75 mM Tris pH 6.8, 3% SDS, 15% glycerol, 8% beta-mercaptoethanol, 0.1% bromophenol blue). All samples were separated by standard SDS-PAGE and transferred onto PVDF membrane (Immobilon-FL). Membranes were blocked with Aquablock tm/EIA/WB (EastCoast Bio) for 1 h, then incubated with primary antibodies to survivin (Cell Signaling), (Thr 68) phospho-Chk2 (Cell Signaling), Chk2 (Cell Signaling), Aurora B kinase (AIM, BD Transduction Labs), MCL1 (Cell Signaling), c-ABL1 (Cell Signaling) and α-tubulin (Cell Signaling) in Aquablock/0.1% Tween-20 overnight at 4°C. Secondary fluorescent antibodies (Molecular Probes) were used and detected with Odyssey (LI-COR).

### Gamma H2AX staining

Staining was performed as described by Muslimovic et al. [25]. Briefly, the cells were treated for 24 h with 100 nM YM155 and then partially fixed with 0.2% paraformaldehyde solution for 5 min and then suspended in Block 9 buffer. The cells were stained with anti-H2AXS139PH FITC (Sigma) conjugate to the final concentration of 0.6 μg/ml in Block-9 staining buffer for 4 h. Vibrant DyeCycle Violet Stain (Molecular Probes) was then added for an additional hour, and the cells were then subjected to flow cytometry with BD FACS/Aria and data interpreted by FlowJo.

### Comet assay

The cells were treated with drug and then processed using CometAssay®HT (Trevigen). Briefly, after treatment, the cells were suspended in low melting agarose and plated on 20-well slides. DNA was denatured using alkaline solution (200 mM sodium hydroxide, 1 mM EDTA) and then underwent electrophoresis. The cells were then fixed with 70% ethanol, dried, and stained with diluted SYBR® Gold, and images were captured by epifluorescence microscopy (Zeiss). Quantitative analysis was performed using Open Comet (http://www.cometbio.org/) [33].

## Additional files

Additional file 1: Figure S1. Dose–response of leukemic samples to YM155. **(A)** A selection of primary samples representing sensitive and resistant samples to YM155. Primary patient samples were treated with increasing concentrations of YM155 and assayed for viability by MTS. Viability was then normalized to no drug control. (#) denotes sample number in the supplemental tables. Black lines represent resistant samples. Red lines represent sensitive samples. **(B)** Ph^+^ BCP-ALL cells show a dose-dependent decrease in survivin and Mcl1 expression when treated with YM155. Xenograft samples from Ph^+^ALL samples 10–668 and10-378 were treated with increasing doses of YM155 (0, 10, 100 nM) for 24 h and harvested. The cell lines HAL01 (YM155 insensitive) and SUPB15 were also treated as controls. Twenty micrograms of total protein was loaded per well, separated by gel electrophoresis, transferred and blotted for survivin, Mcl1, and tubulin. **(C)** Xenograft samples from Ph^+^ALL samples 10–378, BLQ5, SFO2, and TXL3 were treated with 1 μM YM155. Additional file 2:
**Table S1 and S2 ALL patient samples.**
