# Supplementary material for: YM155 potently kills acute lymphoblastic leukemia cells through activation of the DNA damage pathway
Source: J Hematol Oncol. 2015 Apr 22;8:39. doi: 10.1186/s13045-015-0132-6 (PMC4408565; doi:10.1186/s13045-015-0132-6)
Supplement: Additional file 2: — Table S1 and S2 ALL patient samples. [file 13045_2015_132_MOESM2_ESM.docx]

Table 1: ALL Patient Samples

| Number | Disease | Age (yr) | NCI Risk | IC50 |
| --- | --- | --- | --- | --- |
| 1 | B-ALL | 3 | HR | >1000 |
| 2 | B-ALL | 4 | HR | >1000 |
| 3 | B-ALL | nd | nd | 16.3 |
| 4 | B-ALL | 40 | na | 684.9 |
| 5 | B-ALL | 40 | na | 10.6 |
| 6 | B-ALL | nd | nd | 3.9 |
| 7 | B-ALL | nd | nd | 10.1 |
| 8 | B-ALL (1;19) | 11 | HR | 576.4 |
| 9 | B-ALL (11q23) | 3 | SR | 57.8 |
| 10 | B-ALL (11q23) | 34 | na | 684.9 |
| 11 | B-ALL (11q23) | 20 | na | >1000 |
| 12 | B-ALL (11q23) | 43 | na | 31.0 |
| 13 | B-ALL (11q23) | 43 | na | >1000 |
| 14 | B-ALL (11q23) | 0.75 | na | >1000 |
| 15 | B-ALL (11q23) | 24 | na | 34.8 |
| 16 | B-ALL (12;21) | 6 | HR | 16.8 |
| 17 | B-ALL (12;21) | 5 | SR | 476.8 |
| 18 | B-ALL (12;21) | 4 | SR | 43.4 |
| 19 | B-ALL (9;22) | 6 | HR | 10.1 |
| 20 | B-ALL (9;22) | nd | nd | >1000 |
| 21 | B-ALL (9;22) | X | nd | 8.2 |
| 22 | B-ALL (9;22) | X | nd | 20.4 |
| 23 | B-ALL (9;22) | X | nd | 14.5 |
| 24 | B-ALL (9;22) | X | nd | 721.2 |
| 25 | B-ALL (9;22) | 54 | na | 43.4 |
| 26 | B-ALL (9;22) | 52 | na | 3.9 |
| 27 | B-ALL (9;22) | X | nd | 4.2 |
| 28 | B-ALL (9;22) | X | nd | 4.5 |
| 29 | B-ALL (HD) | 3 | HR | 10.6 |
| 30 | B-ALL (HD) | 3 | SR | 310.0 |
| 31 | B-ALL (HD) | 5 | SR | >1000 |
| 32 | B-ALL (HD) | 4 | SR | 684.9 |
| 33 | B-ALL (HD) | 8 | SR | 368.2 |
| 34 | B-ALL (HD) | 23 | na | 21.1 |
| 35 | B-ALL (HD) | 41 | na | 9.9 |
| 36 | B-ALL (HypoD) | 14 | HR | 27.1 |
| 37 | B-ALL (HypoD) | 54 | na | 15.2 |
| 38 | B-ALL (HypoD) | X | nd | 46.4 |
| 39 | B-ALL (HypoD) | X | nd | >1000 |
| 40 | B-ALL (HypoD) | X | nd | 799.9 |
| 41 | B-ALL (HypoD) | X | nd | 110.6 |
| 42 | B-ALL (HypoD) | X | nd | 45.6 |

nd: no data available

na: not applicable (either age <1 year or adult, therefore not NCI risk)

Table 2: AML Patient samples

| Number | Disease | Age | Risk | IC50 |
| --- | --- | --- | --- | --- |
| 1 | AML | 13 | 11q23 | 733.8 |
| 2 | AML | 45 | 11q23 | >1000 |
| 3 | AML | 79 | 2ndary | >1000 |
| 4 | AML | 80 | FLT3-D835 | >1000 |
| 5 | AML | 74 | FLT3-D835 | >1000 |
| 6 | AML | 56 | FLT3-D835 | >1000 |
| 7 | AML | 54 | FLT3-D835 | 61.9 |
| 8 | AML | 65 | FLT3-D835 | 786.2 |
| 9 | AML | 61 | FLT3-D835 | 445.1 |
| 10 | AML | 59 | FLT3-D835 | 415.4 |
| 11 | AML | 51 | FLT3-ITD | 145.5 |
| 12 | AML | 60 | FLT3-ITD | 235.4 |
| 13 | AML | 36 | FLT3-ITD | 576.4 |
| 14 | AML | 66 | FLT3-ITD | >1000 |
| 15 | AML | 9 | FLT3-ITD | 759.5 |
| 16 | AML | 54 | FLT3-ITD | 708.9 |
| 17 | AML | 71 | FLT3-ITD | >1000 |
| 18 | AML | 57 | FLT3-ITD | 452.8 |
| 19 | AML | 37 | FLT3-ITD | 528.8 |
| 20 | AML | 62 | FLT3-ITD | >1000 |
| 21 | AML | 72 | FLT3-ITD | >1000 |
| 22 | AML | 59 | FLT3-ITD | >1000 |
| 23 | AML | 64 | FLT3-ITD | 721.2 |
| 24 | AML | 28 | FLT3-ITD | >1000 |
| 25 | AML | 56 | FLT3-ITD | >1000 |
| 26 | AML | 34 | FLT3-ITD | >1000 |
| 27 | AML | 65 | INV16 | 315.4 |
| 28 | AML | 59 | INV16 | 460.7 |
| 29 | AML | 72 | monsomy 7 | 310.0 |
| 30 | AML | 49 | monsomy 7 | >1000 |
| 31 | AML | 35 | npm+ | 114.5 |
| 32 | AML | 48 | npm+ | 59.8 |
| 33 | AML | 60 | npm+ | 950.5 |
| 34 | AML | 62 | npm+ | 476.8 |
| 35 | AML | 35 | npm+ | 178.8 |
| 36 | AML | 50 | npm+ | >1000 |
| 37 | AML | 61 | rx related | 172.7 |
| 38 | AML | 31 | t(16;16) | 3.9 |
| 39 | AML | 46 | t(8;21) | 361.9 |
| 40 | AML | 72 | NR | >1000 |
| 41 | AML | 58 | NR | 445.1 |
| 42 | AML | 79 | NR | >1000 |
| 43 | AML | 80 | NR | 528.8 |
| 44 | AML | 60 | NR | 538.0 |
| 45 | AML | 36 | NR | 918.2 |
| 46 | AML | 77 | NR | 326.4 |
| 47 | AML | 82 | NR | >1000 |
| 48 | AML | 61 | NR | >1000 |
| 49 | AML | 70 | NR | 227.4 |
| 50 | AML | 81 | NR | 437.5 |
| 51 | AML | 81 | NR | >1000 |
| 52 | AML | 50 | NR | 708.9 |
| 53 | AML | 54 | NR | >1000 |
| 54 | AML | 18 | NR | 430.0 |
| 55 | AML | 70 | NR | >1000 |
| 56 | AML | 81 | NR | >1000 |
| 57 | AML | 54 | NR | >1000 |
| 58 | AML | 54 | NR | 650.3 |
| 59 | AML | 57 | NR | 387.8 |
| 60 | AML | 78 | NR | >1000 |
| 61 | AML | 70 | NR | 576.4 |
| 62 | AML | 69 | NR | >1000 |
| 63 | AML | 41 | NR | >1000 |
| 64 | AML | 72 | NR | >1000 |
| 65 | AML | 51 | NR | >1000 |
| 66 | AML | 24 | NR | >1000 |
| 67 | AML | 68 | NR | >1000 |
| 68 | AML | 41 | NR | >1000 |
| 69 | AML | 45 | NR | >1000 |

NR: no risk feature
